# Impact of COVID-19 Pandemic on Willingness to Consume Insect-Based Food Products in Catalonia

**DOI:** 10.3390/foods10040805

**Published:** 2021-04-08

**Authors:** Reine Khalil, Zein Kallas, Amira Haddarah, Fawaz El Omar, Montserrat Pujolà

**Affiliations:** 1 Departament d’Enginyeria Agroalimentària i Biotecnologia, Campus del Baix Llobregat, Universitat Politècnica de Catalunya, BarcelonaTech, Carrer Esteve Terradas 8, 08860 Barcelona, Spain; montserrat.pujola@upc.edu; 2 Doctoral School of Sciences and Technology, Rafic Hariri Campus, Lebanese University, Hadath 1003, Lebanon; amira.haddarah@ul.edu.lb (A.H.); fomar@ul.edu.lb (F.E.O.); 3 CREDA-UPC-IRTA, Center for Agro-food Economics & Development, Carrer Esteve Terradas 8, 08860 Barcelona, Spain

**Keywords:** consumer acceptance, COVID-19, edible insects, food, sustainability

## Abstract

Edible insects are being considered as a sustainable source of protein and are continuously appearing in markets in the West. The impact of COVID-19 on the willingness to consume (WTC) two products enriched with insect ingredients, jam and yogurt, was analyzed. A semistructured questionnaire was applied using the Qualtrics© consumer panel. Data was collected from 799 and 481 consumers before and during the COVID-19 lockdown in Catalonia (Spain), respectively. The multinomial logit (MNL) model was used to analyze the determinant factors affecting consumers’ WTC insect-based products and the impact of COVID-19 on such heterogeneity. Results showed that the outbreak of COVID-19 caused a significant decrease in the WTC. Findings also revealed that consumers who contracted the COVID-19, strictly followed the regulations during the confinement, and are well informed about symptoms were more likely to reject the consumption of the insect-based products. Both before and during the lockdown, results showed that young and employed consumers, with low-income level, who give importance to the environmental attribute in food are prone to consume insect-based food products. The COVID-19 outbreak had a homogenizing impact on consumers’ WTC with respect to the gender variable. Consumers’ affirmation towards strict food safety standards of the insect-based products should be remarked.

## 1. Introduction

Food and drinks enriched with insects as ingredients have started appearing on the European market since 2014 and have significantly increased since 2018. According to the Global New Products Database (GNPD) of Mintel©, the number of products launched in the European market based on insect compound reached 82 novel products to date. While some of these products are presented in whole insects’ format, the majority of the innovative products include the insects in a flour format as an additional ingredient in products like pasta, bread, burger patties, energy bars, crackers, granola, protein shakes, and spreads [1].

The inclusion of insects as an “invisible” ingredient in familiar products may increase their acceptance by Western consumers, where insects are still considered an unusual and novel food [2]. The insects used in these products are mainly crickets (*Acheta domesticus*) and mealworms (*Tenebrio molitor*) [1]. In 2019, about 9 million consumers in Europe consumed insects and insect-based products and this number is expected to reach 390 million consumers by 2030 [3]. This forecast is expected after the authorization of insects in Europe which will lead to an increased diversity and availability of insect products at market place, and its inclusion in future dietary trends [3].

Insect consumption is very common worldwide such as in Southeast Asia, the Pacific, sub-Saharan Africa, and Latin America [4] while in Europe, whole insects and their parts are a novel food since there is no significant history of its consumption before 15 May 1997 [5]. Nevertheless, with the increased environmental awareness around the world, edible insects have been gaining more attention from research centers and the food industry. They are considered a sustainable source of protein compared to conventional animal protein sources such as beef, pork, and chicken. The worldwide growing demand for meat products [6] associated to an increase in the human population and income growth [7] is reducing the sustainability of the current animal production systems. In 2017, the global demand for meat was estimated at 323 million tons and is expected to increase by 15% in 2027 [8]. Meat is consumed the most in high-income countries. However, meat consumption per capita in these countries has been more or less stable while the major increase in consumption is taking place in countries that are witnessing an enhanced standard of living [9].

The livestock sector generates 9–25% of total Global Greenhouse Gas (GHG) emissions with differences mainly due to different calculation processes [10]. The industrial farming of livestock has a negative impact on biodiversity and habitats, and on water and land resources [11,12]. As for the health effects, the consumption of meat frequently and in high amounts, increases the risk of developing cardiovascular diseases and obesity [13].

On the contrary, insect rearing is sustainable considering its low water and land consumption, low greenhouse gas emissions, and high feed conversion efficiency compared to conventional livestock in particular cattle [14]. These benefits are promoted on some insect products already placed at the market level to encourage consumption. On the other hand, insects are in general considered a good source of protein with a very attractive amino acid profile. They provide a variety of minerals and vitamins and are rich in fat—in particular unsaturated fatty acids [2]. However, it is worthy to note that this good nutrient profile depends on the insect species, the stage of development, and the type of feed [4].

Regardless of the benefits, the edible insect sector in Europe struggles with low consumer acceptance due to consumers’ food neophobia trait, feelings of disgust, cultural inappropriateness, association of insect-eating with primitive behavior, and lack of knowledge about entomophagy [2,13,15]. Another important limitation for its consumption is that insects are viewed as vectors of zoonotic diseases that are harmful to humans such as malaria, yellow fever, and dengue [16,17] and as dirty pests rather than as a nutritious food item [14].

This image is currently even more pronounced with the spread of the infectious novel coronavirus disease, COVID-19, throughout the world. On 11 March 2020, WHO characterized the COVID-19 outbreak as a pandemic after it has spread to 114 countries [18]. As a result, a potential shift in the food choices of consumers might occur, in particular concerning food of animal origin. Therefore, it is expected that there will be a higher rejection to consume unusual foods of animal origin like insects.

It is not surprising that outbreaks of infectious diseases have an impact on consumption patterns of food of animal origin. For example, in 2009, the influenza virus “2009 H1N1” in the United States caused countries like China and Russia to ban the import of pork products [19]. Another example is the Bovine Spongiform Encephalopathy (BSE) crisis in Germany during 2001 which resulted in a 50–80% drop in meat consumption [20].

In the case of insects used for food and feed, there are also concerns about the risk of transmission of zoonotic infections to humans. More research in this area is needed [16]. According to Dossey et al. [4], insects are known to carry viruses and infect humans through direct blood injection; however, it has not been reported that these viruses can be transmitted through ingestion. The European Food Safety Authority (EFSA) conducted a risk assessment for insects used for food and feed and another risk assessment for the house cricket (*Acheta domesticus*). It states that viruses are a low-risk hazard for humans and vertebrate animals [21]. Most insect viruses are targeted at specific families and even specific species of insects and cannot cross to vertebrates. They only cause production losses to farmers. The only risk is that there is evidence that vertebrate viruses can be carried by insects if introduced by the feed and therefore there might be a risk of transmission to humans. This risk could be controlled by selecting certified substrates and ensuring proper processing of the insects [22]. Concerning the current pandemic, the recent article of Dicke et al. [23] stated that the novel severe acute respiratory syndrome coronavirus 2 (SARS-CoV-2) responsible for COVID-19 cannot replicate in insects.

In this study, two insect-based novel products are explored: strawberry jam and the Greek-type natural yogurt. Based on the review of Melgar-lalanne et al. [24] and Mintel© GNPD Database, these products are still not available at the market and thus, could be considered as new products. In Europe and in particular, in Spain, jam is consumed on a daily basis, mostly for breakfast [25]. As for yogurt, it is also a frequently consumed product and offers diverse choices for consumers such as yogurt high in protein or high in fiber. Similarly, now there is demand for sweet spreads with added nutrition and health claims [1]. Adding insect ingredients to these products can provide such claims since they are rich in protein and omega 3 fatty acids [13]. They are also rich in micronutrients like copper, iron, zinc, and vitamin B12 [4] which support the immune system [26].

A large number of research articles studied consumers’ attitudes towards insects in different countries through surveys and tasting sessions. Some of them include the study of Gere et al. [2] which revealed that Hungarian consumers who are willing to reduce their meat intake showed an increase in the number of insect types they would eat as a substitute for meat. This is similar to the findings of Verbeke [15] which defined adventurous young males that are interested in the environmental impact of their food choices and have a weak attachment to meat as potential adopters of insects as meat substitutes. Italian consumers’ acceptance of insect food was also studied by Cicatiello et al. [27]. The results revealed that consumers who attend ethnic restaurants more frequently and have higher education levels are more likely to consume insects. Furthermore, Woolf et al. [28] highlighted the importance of familiarity and exposure to insects in increasing the willingness to consume insect-based food in the US. Additionally, participants that knew two or three benefits about insect-eating (nutritional, environmental, economical, good flavor, and other types of benefits) had a higher willingness to consume.

The main objectives of this research were to investigate whether the outbreak of COVID-19 will have an impact on consumers’ willingness to consume the two insect-based products and to understand the factors affecting the willingness to consume (WTC) shift if it exists. To our knowledge, this is the first study to determine the effect of COVID-19 lockdown on consumers’ expected acceptance of insect-based products. Secondly, to analyze the expected willingness to consume two new products with insect ingredients, strawberry jam and yogurt, not yet available on the market. Thirdly, to analyze the effect of sociodemographics and food consumption/purchasing behaviors and attitudes on willingness to consume insect-based products in Catalonia (Spain).

## 2. Materials and Methods

### 2.1. Data Collection and Sampling Method

Data was collected in Catalonia (Spain) from two waves using Qualtrics© platform and its consumer online panel. A pretest was run by 25 people in each wave. The target of the sample was consumers who regularly purchased food and are residents in Catalonia. The first wave of data was collected from 799 (two respondents below the age of 18 years old and one respondent above the age of 79 years old were excluded, respectively, from wave 1 and wave 2 since they are not in the target age group of our study) participants between January and February 2020 before the COVID-19 lockdown in Catalonia. The second wave of data was collected from another 4811 participants between 21 May and 2 June 2020 during the COVID-19 lockdown in Catalonia. The questionnaire was available in two languages, Catalan and Spanish. The sample was stratified by gender, age, and location in Catalonia to ensure a representative sample of the population.

In Table 1, a summary of the sample description is represented. The experiment was approved by the ethics committee of the Center for Agro-food Economics and Development (CREDA) and was conducted according to the ethical principles with specific care on protecting personal information according to the national and European regulations.

### 2.2. Questionnaire Design

The semistructured questionnaire consisted of three blocks. The first part included questions about consumers’ sociodemographics: age, gender, education level, income, employment situation, and family size among other variables. The expected willingness to consume (WTC) question of strawberry jam and natural yogurt enriched with insect protein constituted the second part. The last part was related to consumers’ food consumption/purchasing behavior opinions and attitudes. Several questions were introduced: the food purchase place and consumers’ relative importance of the different product attributes (price, origin, quality, convenience, nutritional value, and ecological value when buying food). In addition, this part included questions about consumers’ opinions and attitudes towards sustainable and environmental behavior. In the second wave, the questions of this part were more related to the impact of COVID-19 on consumers’ consumption and purchasing behaviors. There were additional questions related to the current health concern, risk behavior, if they have contracted COVID-19 or know someone that did, how strictly they are following restrictions, how well they can identify symptoms of COVID-19, and containment measures in place among other variables.

### 2.3. Consumers’ WTC the Proposed Edible Insect-Based Products

Respondents had to choose their willingness to consume from a five categorical alternatives scale (yes, probably yes, do not know, probably no, and no) on a hypothetical fixed sensory condition that organoleptic characteristics will remain unaltered as a result of adding insect protein. This condition, even though hard to achieve in a real product context, was added to avoid consumers’ reluctance associated with the alterations about what the product might look, taste, and smell like. Furthermore, it was also explained that the reason behind such enrichment is based on the fact that insects are an environmentally sustainable source of protein compared to other proteins of animal origin.

An example of the question is presented below:

*The increasing demand for the consumption of meat as a source of protein is compromising the sustainability of the systems of animal production. Products enriched with insect proteins are currently appearing on the Spanish market as an environmentally sustainable source of protein compared to protein of other animal origin. Would you be willing to consume enriched food products with insect protein if their organoleptic characteristics remain unaltered (taste, color, and odor)?*

### 2.4. Determinant Factors Affecting the Consumers’ WTC. The Multinomial Logit (MNL) Model

In order to analyze the determinant factors affecting the consumers’ WTC, the multinomial logit (MNL) model was applied using IBM SPSS (v. 22.0 for Windows; IBM, New York, NY, USA). The MNL is a probabilistic regression model that is used when the dependent variable is categorical and consists of more than two categories. In our case, the dependent variable was the WTC variable (yes, probably yes, I do not know, probably no, and no). The five categories were grouped into three categories: *positive WTC* (yes), *uncertain WTC* (probably yes, do not know, probably no), and *negative WTC* (no).

The independent variables included in the MNL were the socioeconomic variables and the consumers’ behavior variables. Consumers’ risk perception and sustainability and environmental attitudes were also explored for their significance. The dependent variables can be continuous or dichotomous (dummy variables). The model is constructed separately for each category after choosing one category of the WTC as the reference category. According to the MNL model, the probability of consumers to select one of the other two categories (i.e., different from the reference category) can be obtained as follows:

For categories i=2…k of the dependent variable, the probability is:

(1) $$\mathrm{Pr}\;(Y=i)\;=\;\frac{e^{Z_{i}}}{1\;+\;\sum_{h=1}^{H}e^{Z_{hi}}}$$

where $\alpha_{i}+\;\overset{H}{\underset{h=1}{\sum }}\beta_{ih}X_{ih}=\;Z_{i}$.

For the reference category, the probability is:

(2) $$\mathrm{Pr}\;(Y=1)\;=\;\frac{1}{1\;+\;\sum_{h=1}^{H}e^{Z_{hi}}}$$

Rearranging Equations (1) and (2), the MNL can be written as follows:

(3) $$\frac{P\left(Y=i\right)}{P\left(Y=1\right)}\;=\;\exp\;(\alpha_{i}+\;\sum_{h=1}^{H}\beta_{ih}X_{ih})$$

where i is the number of WTC categories under scrutiny, $\alpha_{i}\;$ is a constant, $\beta_{ih}X_{ih}$ are the vectors of the estimates parameters and the predictor variables, respectively, $\frac{P\left(Y=i\right)}{P\left(Y=1\right)}$ is the probability of one prospect to make a choice other than the reference category, and h takes values between 1 and H, H being the number of independent variables included in the model.

For example, for the category “positive WTC” of jam before lockdown, where “negative WTC” is the reference category, the equation would be:

(4) $$\frac{P\left(positiveWTC\right)}{P\left(negativeWTC\right)}\;=\exp\begin{array}{c} \alpha_{\left(positiveWTC\right)}+\beta_{1\;Gender}+\beta_{2\;Age}+\beta_{3\;Internet}\\ (+\beta_{4\;Origin}+\beta_{5\;Ads}+\beta_{6\;Environment}+\beta_{7\;Quality}\\ +\beta_{8\;Labor}+\beta_{9\;Expenditure}) \end{array}$$

where $\beta_{1}$–$\;\beta_{9}$ are the coefficients of each predictor variable, respectively.

Equation (3) expresses the logit (log odds) as a linear function of the independent factors ($X_{ih}$). For each variable, the odds ratio is calculated ${\mathrm{OR}}_{i}=\;e^{\beta_{i}}$ which represents the modification that occurs in the dependent variable for each one-unit increase in the continuous independent variable or change in the category level of a dummy independent variable, ceteris paribus (the other variables in the model held constant).

## 3. Results and Discussion

### 3.1. WTC before and during Lockdown

The descriptive results of the WTC are presented in Table 2. As can be seen, there is a statistically significant difference between the willingness to consume insect-based products before and during the COVID-19 outbreak in Catalonia (*p* < 0.01). During COVID-19, the percentage of participants prone to consume decreased by an average of 5.80% for both products and increased by an average of 12.35% for those reluctant to consume.

It seems that some consumers link the consumption of insect-based products as an unfamiliar product of animal origin with potential virus transmission. This perceived interaction is also mentioned when meat products are consumed [29]. A similar pattern was observed due to the outbreak of the highly pathogenic avian influenza HPAI H5N1 which caused a 40–50% decrease in demand for chicken meat in Italy at the end of 2005 [30] and in Vietnam, 74% of the consumers stopped eating poultry in January 2004 [31]. A pandemic puts people under an immense state of fear and stress especially when it is coupled with financial insecurity. The COVID-19 lockdown changed everyone’s lifestyle with people staying at home and practicing social distancing. Diets also changed with more consumers seeking products with immunological benefits [32]. All of these factors which will be further discussed in the following sections have contributed to this decrease in willingness to consume insects.

The results also indicate that the percentage of participants reluctant to consume (negative WTC) is higher than the percentage of participants prone to consume (positive WTC) which is in line with other studies. The percentage of participants willing to consume insects as an alternative to meat protein was 25% in the study of Gómez-Luciano et al. [9] in Spain, 19.3% in the study of Verbeke [15] in Belgium, and 31% in the study of Cicatiello et al. [27] in Italy. Another study by Roma et al. [33] in Italy revealed that 16.8% of the respondents were willing to consume insect-based products. The willingness to try yogurt with insect protein was previously analyzed by Ardoin and Prinyawiwatku [34] and was estimated at 38.01% which is higher than our results. The differences in results depend on several factors, mainly, on the sample characteristics, the familiarity of insect consumption in each European country and availability of products in the market, the amount of information about edible insects explained in the questionnaire, the form of insects included in the product, and the scale used for measuring willingness to consume among others. However, tasting the products might improve its acceptance by the respondents uncertain or reluctant to consume according to the findings of Tan et al. [35] and Woolf et al. [28].

### 3.2. Factors Affecting the WTC Insect-Based Products

Four MNL models were estimated using the WTC of the proposed products as the dependent variable. Each product (jam and yogurt) has two models, one before the COVID-19 lockdown and another during the COVID-19 lockdown. As mentioned before, the dependent variable has three levels: positive WTC, uncertain WTC, and negative WTC. The independent variables included in the models are presented in Table 3 and Table 4.

Results of the proportional by chance accuracy rate were computed by calculating the proportion of each WTC category. The classification accuracy rate typically should be 25% or more, higher than the proportional by chance accuracy rate. Therefore, the squared proportions of all categories were summed and multiplied by 1.25. Accordingly, the results showed a good model fit.

Results of the estimated MNL models are shown in Table 5 and Table 6. In all models, the null hypothesis that there was no improvement in the model with independent variables compared to the model without independent variables is rejected at 99% confidence level according to the Log-Likelihood ratio test. In addition, multicollinearity was tested. We considered the most widely used diagnostic for multicollinearity, the variance inflation factor (VIF). In all models, the VIF value for all variables was below 2.5 showing the irrelevance of the multicollinearity problem in our logit estimation.

The comparison of the common independent variables between the models before and during lockdown is highlighted first. For simplicity, it will be focused on one product in the case of common variables between the products while the reader can draw out similar results for the other product. In the same line, before COVID-19, the comparison will be carried out between the positive WTC and the negative WTC while the reader can draw out similar results comparing the uncertain WTC to negative WTC. During COVID-19, the positive or uncertain WTC will be compared to the negative WTC depending on the significance of the variable.

#### 3.2.1. Common Determinant Factors of Consuming Insects before and during COVID-19

The independent variables that are common between the two waves, before and during COVID-19 lockdown, are presented and discussed below.

##### Gender

According to the gender variable, before lockdown, males compared to females are 3.23 times more likely to consume jam enriched with insect protein (IP). This is in accordance with other research studies since males are less food neophobic [2,15] and less disgusted by insects [14]. Another reason is that females are more concerned about the safety of food than males [36] and according to Liu et al. [37], consumers are generally skeptical about the safety of edible insects. However, during the lockdown, this gender difference changed. Males compared to females are 1.45 times more likely to be uncertain about their willingness to consume jam enriched with IP rather than negative. The males during lockdown have shifted from a positive willingness to consume to an uncertain behavior and both males and females are not likely to be willing to consume the product. It seems that the COVID-19 outbreak has had a homogenizing effect on the gender variable when analyzing the heterogeneity of the willingness to consume.

##### Age

Before the lockdown, consumers belonging to the age group of 18–39 years old are 3.55 times more likely to consume jam enriched with IP than older age groups. Young consumers have a higher likelihood to accept insects since they are more open to new food choices as also commented in Caparros Megido et al. [38] and Orsi et al. [14]. Furthermore, older consumers are less likely to adopt alternatives to meat such as insects [15] and are more traditional with the food they eat [39]. This effect was maintained during the COVID-19 outbreak where participants of the age group 18–39 years old compared to the older age groups are 4.88 times more likely to consume the jam enriched with IP. Results revealed that the COVID-19 outbreak has increased the willingness to consume heterogeneity between the different age groups.

##### Environmental Respectfulness/Ecological Importance

Consumers before lockdown that consider consuming food produced in Catalonia is more environmentally respectful are 9.02 times more likely to consume yogurt enriched with insects. Results showed that consumers with a higher environmental awareness are more interested in the consumption of insects which is also verified by Kornher et al. [13] and Orsi et al. [14] highlighting its potential as a sustainable source of protein. In the same way, consumers during the lockdown who give higher importance to the environmental attribute when shopping for food are 2.35 times more likely to consume insect-based yogurt. However, the effect size of the environmental factor during COVID-19 is reduced which justifies that an economic crisis may drive people’s attention away from environmental issues [32] as other issues become of higher priority.

##### Labor Situation

Before the lockdown, employed consumers compared to unemployed are 1.87 times more likely to consume yogurt enriched with IP. This could be due to the relation between the economic situation and the ability to experience new food. Torri et al. [39] mentioned that consumers with higher capacity to travel are more exposed to exotic foreign food from different cultures which allows them to be more open to novel food choices. In the same way, Cicatiello et al. [27] found that consumers that attend ethnic restaurants more frequently are more accepting of insect food products. These two activities, traveling and attending ethnic restaurants, help to decrease the food neophobia trait in consumers. Therefore, consumers with a job compared to those that are unemployed have higher chances of traveling, attending ethnic restaurants, and hence, experiencing novel food. Conversely, consumers that are not working and are with a limited budget will focus on the essential food products and will reduce their spending on novelties. During the COVID-19 lockdown, employed consumers are 1.99 more likely to consume yogurt enriched with insects. Borsellino et al. [32] mentioned that during a financial crisis as the case with the COVID-19 pandemic especially with increased unemployment, nonessential spending is reduced. However, the readiness to consume insects during the COVID-19 pandemic was maintained showing that the effect of employment situation was unaltered in defining consumer heterogeneity to consume insects.

##### Income

Consumers before lockdown with a monthly income of EUR 1000 or less compared to those with a higher income are 2.28 times more likely to consume yogurt enriched with IP. Therefore, consumers with the lowest income have shown the highest willingness to consume insects. This could be explained since insects are considered as food consumed by primitive people and associated with poverty [40,41]. Additionally, meat consumption compared to other sources of protein increases with the increase of income [7]. Even in places where insects are traditionally consumed, this practice is in decline due to the Westernization of diets globally [15] and an increase in the demand for meat [7]. In other words, it is like consumers are associating insect consumption with poverty and meat consumption with prestige [12]. The same outcome is also observed during lockdown, however, with increased heterogeneity. Consumers during lockdown with a monthly income of EUR 1000 or less compared to those with a higher income are 4.05 times more likely to consume yogurt enriched with IP. In Oaxaca, Mexico, people from all economic classes spend equally on edible insects [4]. This means that consumers from a lower economic class give a higher importance to insects within their food budget which is in agreement with our results. However, the effect of income on willingness to consume insects is not greatly discussed in previous studies and requires more verification. For example, in the study of Woolf et al. [28], income had no effect on willingness to consume insects regularly. Conversely, in Poland, Orkusz et al. [42] found that people with a higher income are both more willing to include insects in their diets and also consume insects more often than those with a lower income. This is justified by the fact that people with higher incomes have a lower level of food neophobia. It seems that the effect of income on willingness to consume insects varies depending on the food culture and social norms in each country.

#### 3.2.2. Determinant factors of consuming insects before COVID-19

The independent variables that are included in the models only before COVID-19 are presented and discussed below.

##### Buying Food Online

The results before COVID-19 showed that consumers that buy food online are 2.60 times more likely to consume jam enriched with IP. Online food consumers are usually young, also confirmed by Singh and Sailo [43] since they are more involved with internet and technology. As explained before, young consumers are more open to insect consumption. Additionally, since insect products are for now available on online stores more than in real markets, online shoppers could be more familiar with them.

##### Origin

On a scale from 1 to 7, with every one unit increase in the importance consumers give to origin when buying food, the odds of being willing to consume jam enriched with IP decreases 0.77 times. Origin is one of the attributes that consumers consider when choosing a product since it gives a general indication about the quality of the product especially in regard to taste and food safety. For example, French consumers have a higher preference for fish sourced from developed countries rather than fish from developing countries as an indicator of higher quality [44]. Insects are known to be traditionally consumed in Southeast Asia, the Pacific, sub-Saharan Africa, and Latin America [4]. Therefore, consumers in general term have the idea that insects originate from developing countries and in turn, are associated with food safety risks which negatively affects the WTC.

##### Advertisements

On a scale from 1 to 7, with every one unit increase in how much consumers agree that advertisements are necessary, the odds of being willing to consume jam enriched with IP increases 1.28 times. Consumers require added information in the case of novel foods to make a tasting or purchasing decision since novel foods such as edible insects are perceived with a high level of uncertainty [45]. Consumers’ knowledge about the benefits of insect-eating (the nutritional, environmental, economical, and sensory benefits) among others encourages their consumption and purchase [28,46]. Thus, consumers that pay attention to advertisements are more likely to be informed about a novel food which might increase their willingness to consume it.

##### Quality Score

On a scale from 1 to 11, the results reveal that with every one unit increase in the relative importance that consumers give to the quality score associated with seasonal food, they are 0.85 times less likely to consume jam enriched with IP. Food product quality includes aspects such as safety, healthiness, taste, appearance, and popularity [43,47,48]. European consumers generally have negative expectations with respect to the taste of edible insects [35]. They are also thought to be disgusting, not safe for consumption, visually unappealing [14,44], and socially rejected as a food [35]. Therefore, for all of these reasons, insect food products are probably thought to be of low perceived quality [49] and thus, consumers that pay higher attention to food quality are less likely to consume it.

##### Expenditure

Results showed that consumers with a monthly food expenditure within or above average compared to those with a food expenditure below average are 0.46 times less likely to consume insect-based jam. This is likely because food expenditure is higher among consumers having a higher income [50] which are less likely to be willing to consume insects as explained before in the income variable.

##### Education

Consumers with a university education compared to those with a lower level of education are 1.71 times more likely to consume yogurt enriched with insects. This is also verified in the study of Cicatiello et al. [27] and Kornher et al. [13] which may be explained due to higher environmental awareness. Similarly, according to Gómez-Luciano et al. [9] consumers with a higher education show a higher acceptance of alternative sustainable proteins in their diets instead of meat and are more concerned with the healthiness of their food choices.

#### 3.2.3. Determinant Factors of Consuming Insects during the COVID-19 Outbreak

The independent variables that are included in the models only during the COVID-19 are presented and discussed below.

##### Following Restrictions/Informed about Symptoms/Contracted COVID-19

The results during COVID-19 indicate that, on a scale from 1 to 7, with every one unit increase in how strictly a participant is following restrictions to prevent the spread of the virus, the odds of rejecting the consumption of jam enriched with IP increases by 0.82 times. In addition, on a scale from 1 to 7, with every one unit increase in the belief that body ache and muscle pain is a common symptom of COVID-19, the odds of rejecting the consumption of insect-based jam increases by 0.75 times. Finally, participants that have contracted COVID-19 compared to those that have not are 0.14 times more likely to reject the consumption of jam with IP.

These three factors which are an indication of the awareness and concern consumers have towards the virus, increase the likelihood to reject the consumption of unfamiliar products of animal origin like insects. On one hand, insect consumption is already perceived to be associated with viruses and diseases [16] and consumers are skeptical about the safety of insect foods [27]. On the other hand, the outbreak of COVID-19 was linked to the trade of live wild animals [51]. This behavior is similar to the case of BSE and genetically modified organisms (GMO), where the lack of risk assessment and scientific information available to consumers caused a level of uncertainty and fear which eventually lead to a decreased consumption of meat and GMO products, respectively [52].

##### Cereal Consumption

Consumers that increased their consumption of cereals during the pandemic are 2.79 times more likely to consume insect-based jam. According to Borsellino et al. [32], consumers focused on a healthier more sustainable diet during the confinement: a diet low in saturated fat, salt, and sugar and high in antioxidants, fibers, and vitamins that aids in fighting diseases. Therefore, it may be that these consumers have also increased their interest in sustainable sources of protein like insects. Another reason is that bakery products with cereals (pasta, bread, cakes, and cookies) and snacks (crackers and energy bars) are of the most appropriate products to be combined with insect protein according to the study of Ardoin and Prinyawiwatkul [34]. This suggests that there might be a link between consumption of cereals and insects as they share some common flavor characteristics in certain species. For example, mealworms are mostly fed on cereal bran or flour and have a more or less nutty flavor [53].

##### Children at Home

According to the results, during COVID-19, consumers with children of up to 12 years old at home are 2.08 times less likely to reject consuming jam enriched with IP. According to Szendrő et al. [54], children are more interested in insect-based products because possibly they do not focus on what is in the product, but rather on the appearance of the product. Additionally, the study of Zagrobelny et al. [55] revealed that as an old tradition in Carnia, Italy, children would eat the sweet ingluvies (a swollen pocket from the gut) from day-flying moths on flowers. It was explained that children are fascinated by new edible sources in their environment. When children are exposed to insects at a young age, it can be easier to shape their habits and change the negative cultural pre-conception Western consumers have towards insect consumption [56]. However, the question here is directed to respondents with children and not to the children directly. Therefore, more research is needed to explain why this category of consumers shows a higher acceptance towards insect consumption.

##### Increase in Food Prices

Consumers that perceived an increase in food prices during the COVID-19 lockdown are 0.57 times more likely to reject the consumption of insect-based jam. A financial crisis like the one witnessed during this pandemic leads to increased market prices, unemployment, and reduced incomes. Hence, people will have to limit their budget to essentials and exclude novel foods, insects in this case, from their shopping list [32].

Finally, a few comments will be added to the discussion. There is a possibility that these differences are due to a bias among the choice of samples and not due to an actual impact of the pandemic situation. However, considering that both samples are stratified by age, gender, and location to ensure a representative sample of the population in Catalonia and are large enough (799 and 481 respondents for first and second sample, respectively), the differences between samples are minimized. In fact, statistical tests were carried out that showed a nonsignificant difference in terms of gender, age, and education (*p* > 0.05).

In addition, the models of the two products belonging to the same wave, whether before or during lockdown, share the majority of the variables that explain the heterogeneity except for one or two variables. Additionally, more or less, the effect size (odds ratio) for each variable is similar between the two products. In the models before lockdown, the variable “food expenditure” is specific to jam, while “education” and “income” are specific to yogurt. In the models during lockdown, the variables “increase in food prices” and “contracted COVID-19” are specific to jam, while “labor situation” and “income” are specific to yogurt. This means that participants are likely focusing on the content of insects in the product in general and not on the type of product combined with insects. Further tasting experiences are required to allow the differentiation between the product combinations. Besides, a real scenario where products are actually offered to the prospects will reduce hypothetical bias. In this study, a cheap talk script was used at the beginning of the questionnaire to reduce this challenge. The script explained the purpose of the study and highlighted the importance of responding truthfully to the questions in the survey.

## 4. Conclusions

The evaluation of the surveys confirms that the desire to consume insect-based products (for example yogurt and jam enriched with insect protein) during the COVID-19 lockdown decreased significantly and that the gender effect was homogenized. The results also indicate that occidental culture continues to associate insect-based foods as low-quality products and are usually related to feeding low-income countries. Another important finding is that the COVID-19 pandemic has increased population concerns about virus transmission and food safety risks associated with the consumption of insects. Hence, consumers need to be ensured that insect consumption is safe. Product labels should be reinforced with food safety certifications and relevant information such as the origin of insects to gain the trust of potential consumers.

In general, the outbreaks of infectious diseases, COVID-19 among them, have temporary consequences on consumer behavior. A change in trends would be expected which should be confirmed with longer-term research.

## Figures and Tables

**Table 1:** Sociodemographic variables summary.

Variable	Level	Before Lockdown	During Lockdown
Gender	Male	50.9%	49.5%
	Female	49.1%	50.5%
Age group	18–39 years	35.8%	33.3%
	40–59 years	41.9%	38.3%
	60–79 years	22.3%	28.5%
Residence area	Urban	85.0%	90.2%
	Rural	15.0%	9.8%
Education level	Incomplete primary education	1.0%	0.6%
	Primary education	6.6%	4.4%
	Secondary education	40.7%	39.3%
	University education	51.7%	55.7%

**Table 2:** Willingness to consume insect-based strawberry jam and yogurt before and during lockdown.

Product	Event	Positive WTC	Uncertain WTC	Negative WTC	Chi-Square Value	*p* Value
Strawberry Jam	Before lockdown	13.4%	59.9%	26.7%	25.89	0.00
	During lockdown	8.5%	51.8%	39.7%		
Yogurt	Before lockdown	15.8%	58.2%	26.0%	27.43	0.00
	During lockdown	9.1%	52.2%	38.7%		

**Table 3:** Independent variables included in the multinomial logit (MNL) models before lockdown.

Variable Acronym and Description
**V_1_**: Gender: female (0); male (1)
**V_2_**: Age: 40–79 years old (0); 18–39 years old (1)
**V_3_**: Buy food from internet: no (0); yes (1)
**V_4_**: Importance of origin when buying food: not important (1) to very important (7); mean = 4.71, Sd = 1.66
**V_5_**: Ads are a necessary thing in society: totally disagree (1) to totally agree (7); mean = 4.52, Sd = 1.66
**V_6_**: Environmental respectfulness of food produced in Catalonia: lower environmental respectfulness (0); higher environmental respectfulness (1)
**V_7_**: Quality score of seasonal food: lowest quality (1) to highest quality (11); mean = 9.44, Sd = 1.72
**V_8_**: Labor situation: unemployed (0); employed (1)
**V_9_**: Household’s monthly net income: more than EUR 1000 (0); EUR 1000 or less (1)
**V_10_**: Average monthly expenditure on food relative to average (excluding restaurants): below average (0); within or above average (1)
**V_11_**: Education level: below primary, primary, or secondary education (0); university education (1)

**Table 4:** Independent variables used in the MNL models during lockdown.

Variable Acronym and Description
**V_1_**: Gender: female (0); male (1)
**V_2_**: Age: 40–79 years old (0); 18–39 years old (1)
**V_3_**: How strictly have you been following the restrictions to prevent the spread of COVID-19: not strictly (1) to very strictly (7); mean = 6.24, Sd = 1.05
**V_4_**: How has COVID-19 impacted your consumption of cereals: I never consume it, decreased, or did not change (0); increased (1)
**V_5_**: Children at home from 0 to 12 years: no (0); yes (1)
**V_6_**: Body ache or muscle pain is a common symptom of COVID-19: definitely false (1) to definitely true (7); mean = 5.64, Sd = 1.34
**V_7_**: Household’s monthly net income: more than EUR 1000 (0); EUR 1000 or less (1)
**V_8_**: How did the importance of ecological attribute change for you when shopping for food during COVID-19: I never consider it, decreased, or did not change (0); increased (1)
**V_9_**: Did you contract COVID-19 virus: no (0); yes (1)
**V_10_**: Have you experienced an increase in food prices during the COVID-19 outbreak: no (0); yes (1)
**V_11_**: Labor situation: unemployed (0); employed (1)

**Table 5:** Factors affecting willingness to consume (WTC) jam and yogurt enriched with insect protein before COVID-19 lockdown in Catalonia.

	Jam				Yogurt			
	Positive WTC		Uncertain WTC		Positive WTC		Uncertain WTC	
	β	*e^β^*	β	*e^β^*	β	*e^β^*	β	*e^β^*
*α_i_*	−1.55		2.06 ***		−1.27		1.91 ***	
V_1_	1.17 ***	3.23	1.05 ***	2.87	1.06 ***	2.89	1.00 ***	2.72
V_2_	1.27 ***	3.55	0.51 **	1.66	1.00 ***	2.72	0.28	1.32
V_3_	0.95 ***	2.60	0.27	1.31	0.70 **	2.02	0.50 **	1.65
V_4_	−0.27 ***	0.77	−0.22 ***	0.81	−0.29 ***	0.75	−0.25 ***	0.78
V_5_	0.25 ***	1.28	0.11 *	1.11	0.21 ***	1.24	0.14 **	1.16
V_6_	1.60 ***	4.94	1.24 ***	3.44	2.20 ***	9.02	1.25 ***	3.48
V_7_	−0.16 *	0.85	−0.26 ***	0.77	−0.27 ***	0.77	−0.24 ***	0.78
V_8_	0.78 **	2.19	0.25	1.29	0.63 **	1.87	0.34 *	1.41
V_9_	-	-	-	-	0.83 **	2.28	0.10	1.10
V_10_	−0.77 **	0.46	−0.03	0.97	-	-	-	-
V_11_	-	-	-	-	0.54 **	1.71	−0.09	0.91
	McFadden R^2^: 0.118 Class.: 63.7%				McFadden R^2^: 0.113 Class.: 62.2%			

Class. (Classification accuracy rate); significance levels: *** *p* < 0.01, ** *p* < 0.05, * *p* < 0.10. Variable acronyms are explained in Table 3.

**Table 6:** Factors affecting WTC of jam and yogurt enriched with insect protein during COVID-19 lockdown in Catalonia.

	Jam				Yogurt			
	Positive WTC		Uncertain WTC		Positive WTC		Uncertain WTC	
	β	*e^β^*	β	*e^β^*	β	*e^β^*	β	*e^β^*
*α_i_*	−2.88 *		3.26 ***		−1.67		3.09 ***	
V_1_	0.27	1.31	0.37 *	1.45	0.13	1.14	0.42 **	1.52
V_2_	1.59 ***	4.88	−0.24	0.79	1.49 ***	4.42	−0.09	0.91
V_3_	−0.11	0.89	−0.19 *	0.82	−0.25	0.78	−0.26 **	0.77
V_4_	1.03 **	2.79	0.39	1.48	0.94 **	2.55	0.32	1.38
V_5_	0.46	1.58	0.73 ***	2.08	0.34	1.41	0.44 *	1.55
V_6_	0.21	1.24	−0.28 ***	0.75	0.13	1.14	−0.29 ***	0.75
V_7_	1.07 **	2.91	−0.38	0.68	1.40 **	4.06	−0.28	0.75
V_8_	-	-	-	-	0.85 **	2.35	0.16	1.17
V_9_	1.41	4.10	−1.98 *	0.14	-	-	-	-
V_10_	0.03	1.03	−0.56 ***	0.57	-	-	-	-
V_11_	-	-	-	-	0.69 *	1.99	0.30	1.35
	McFadden R^2^: 0.106 Class.: 61.5%				McFadden R^2^: 0.100 Class.: 60.7%			

Class. (Classification accuracy rate); significance levels: *** *p* < 0.01, ** *p* < 0.05, * *p* < 0.1. Variable acronyms are explained in Table 4.
